# Anomalous Coronary Venous Drainage to the Right Atrium: A Case Report

**DOI:** 10.7759/cureus.107737

**Published:** 2026-04-26

**Authors:** Eusebio Luna, Aman Aher, Rohit Reddy, Cibele Luna, Pritish Aher

**Affiliations:** 1 Radiology, University of Miami/Jackson Memorial Hospital, Miami, USA; 2 Nutrition and Exercise Physiology, University of Missouri, Columbia, USA

**Keywords:** anomalous venous drainage, cardiac cta, cardiac veins, coronary sinus, left marginal vein, plsvc.

## Abstract

Anomalies of the coronary venous system are rare and may have important implications for cardiac imaging and invasive procedures. We report a case of anomalous coronary venous drainage identified on chest computed tomography angiography (CTA) in a 53-year-old man undergoing routine follow-up after surgical repair of a type A aortic dissection. Imaging demonstrated a dilated coronary sinus, secondary to distal narrowing, with dilation of the great cardiac vein suggestive of compensatory redistribution of venous return. Additionally, the left marginal vein was observed to drain directly into the right atrium, bypassing the coronary sinus.

An incidental persistent left superior vena cava draining the coronary sinus into the left brachiocephalic vein was also identified, with a normal right superior vena cava present. The patient was asymptomatic, and no intervention was required.

This case highlights a rare variant of coronary venous anatomy, with potential implications for central venous access, cardiac device implantation, and surgical planning. Recognition of such anomalies through careful cross-sectional imaging is essential to avoid diagnostic pitfalls and to ensure safe procedural management.

## Introduction

The normal coronary venous system consists of cardiac veins that drain deoxygenated blood from the myocardium into the coronary sinus, which subsequently empties into the right atrium. Anomalies of the coronary venous system are rare, with an estimated prevalence of less than 1% in the general population, while specific variants involving direct drainage of coronary veins into cardiac chambers are exceedingly uncommon and largely limited to isolated case reports [1-2]. These atypical drainage patterns may complicate anatomical interpretation and procedural planning. Altered coronary venous return can present challenges during interventions, including central venous access, cardiac device implantation, and cardiothoracic surgery, where unrecognized variants may increase the risk of technical difficulty or complications [3-4]. Although often asymptomatic and incidentally detected, these anomalies may occasionally present with angina, arrhythmias, or syncope, particularly when associated with altered venous drainage or coexisting structural abnormalities [5].

Advances in cross-sectional imaging, particularly cardiac computed tomography angiography, have significantly improved the detection and characterization of coronary venous anatomy [2, 5]. Multiplanar reconstruction and three-dimensional visualization enable detailed assessment of venous pathways, facilitating identification of atypical drainage patterns that may otherwise be overlooked [6-7]. This is particularly important in patients undergoing pre-procedural evaluation, where unrecognized venous variants may increase the risk of technical difficulty or complications. A systematic approach to reviewing venous structures is therefore essential to ensure accurate diagnosis and to guide safe and effective intervention [5].

We report a case of anomalous coronary venous drainage, identified on computed tomography angiography chest, with persistent left superior vena cava, emphasizing the importance of recognizing rare coronary venous variants in clinical practice.

## Case presentation

A 53-year-old man presented for routine follow-up 13 months after emergency repair of a type A aortic dissection. He was asymptomatic, with stable vital signs (respiratory rate 14 breaths/min, heart rate 80 bpm, blood pressure 120/85 mmHg), and denied chest pain, dyspnea, palpitations, or exercise intolerance.

His medical history was significant for long-standing hypertension. Thirteen months earlier, he had presented with acute epigastric pain and neurologic symptoms and was found to have an extensive Type A aortic dissection extending from the aortic root to the renal arteries. He underwent emergent surgical repair with ascending aortic replacement, aortic valve resuspension, and extended hemiarch reconstruction, with subsequent recovery.

Investigations

Electrocardiogram-gated cardiac computed tomography angiography (CCTA) was performed for routine postoperative surveillance. The ascending aortic graft was patent, and the residual dissection remained stable.

**Figure 1:**
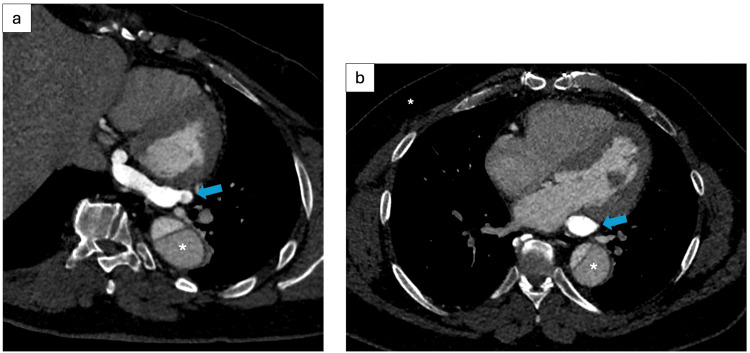
Computed Tomography Imaging of Dilated Coronary Sinus With Early Opacification (a) Axial contrast-enhanced reconstruction along the coronary sinus and (b) axial computed tomography image of the chest demonstrate early contrast opacification of a dilated coronary sinus (arrow), without opacification of the right atrium or contrast mixing, suggesting ostial stenosis. Additionally, a dissection flap is noted in the descending aorta (*).

**Figure 2:**
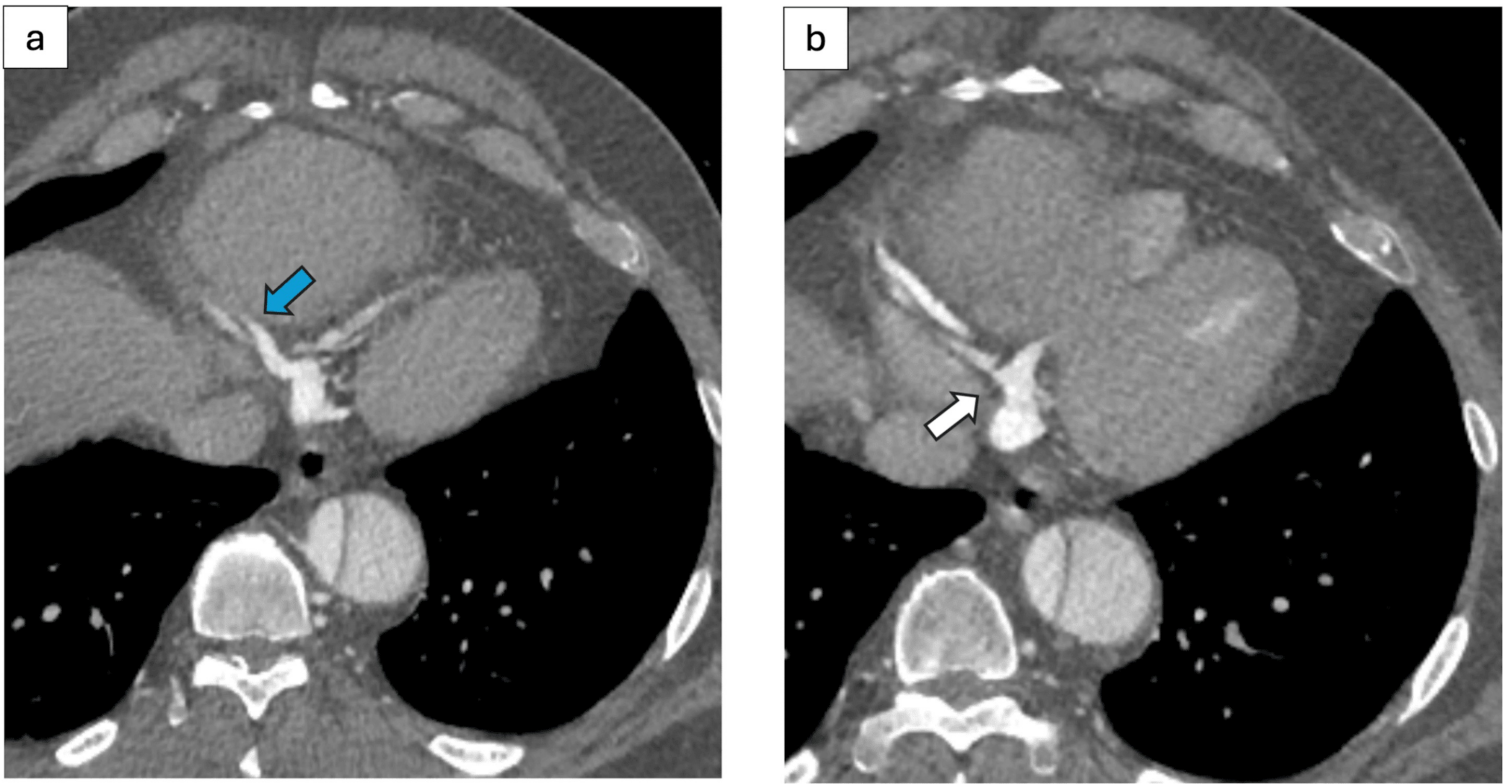
Axial Computed Tomography Images Showing Dilated Right Cardiac Vein and Coronary Sinus Ostial Narrowing (a) Axial contrast-enhanced computed tomography shows dilated right cardiac vein (blue arrow), and (b) axial contrast-enhanced computed tomography at the level of the inferior heart demonstrates coronary artery ostial narrowing (white arrow).

Detailed evaluation of the coronary venous system demonstrated the absence of right atrial opacification or contrast mixing, raising suspicion for ostial stenosis. Imaging revealed an anomalous drainage pattern with focal narrowing of the distal coronary sinus near its ostium into the right atrium (Figures 1-2). This was associated with dilation of the great cardiac vein, likely reflecting compensatory redistribution of coronary venous return. The left marginal vein was also observed to drain directly into the right atrium, bypassing the coronary sinus.

**Figure 3:**
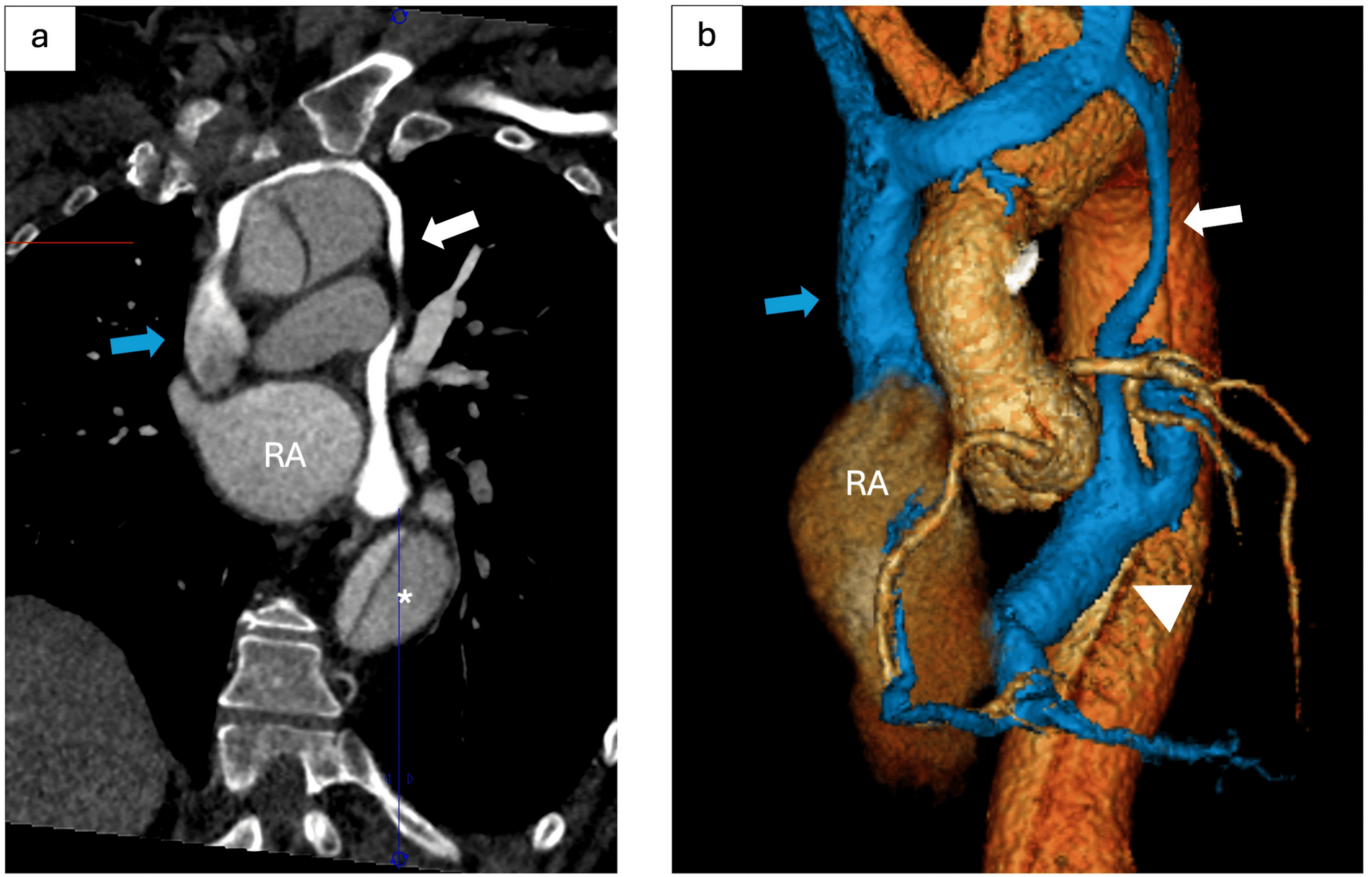
Persistent Left Superior Vena Cava Draining Into the Coronary Sinus on Computed Tomography and Three-Dimensional Reconstruction (a) Curved coronal reconstruction of contrast-enhanced computed tomography of the chest and (b) three-dimensional volume rendering reconstruction of the great vessels demonstrate a persistent left superior vena cava (PLSVC) (white arrow) draining into the coronary sinus (arrowhead). Right SVC (blue arrow) drains normally into the right atrium (RA). Partially visualized dissection flap in the descending aorta (*).

A persistent left superior vena cava (PLSVC) was identified, with altered venous drainage. In the presence of a PLSVC and a normally patent coronary sinus, venous return from the left upper extremity typically drains into the coronary sinus. In this case, however, distal coronary sinus narrowing likely resulted in increased venous pressure, leading to reversal of flow, with decompression from the coronary sinus through the PLSVC into the innominate vein and subsequently into the right superior vena cava and right atrium (Figure 3).

The right superior vena cava was present and demonstrated normal drainage into the right atrium. Other findings included mild narrowing of the right subclavian vein, likely related to prior central venous access, and an incidental 5-mm pulmonary nodule in the left lower lobe.

Transthoracic echocardiography demonstrated preserved left ventricular systolic function (ejection fraction 70%-75%), mild biatrial dilation, and a dilated coronary sinus, without evidence of ventricular dysfunction.

Differential diagnosis

The differential diagnosis for this venous configuration included coronary artery fistula, unroofed coronary sinus, and other anomalous coronary venous drainage patterns. Partial anomalous pulmonary venous return was also considered but excluded based on imaging findings.

Multiplanar and three-dimensional reconstructions confirmed anomalous coronary venous anatomy.

Management and outcome

Given the absence of symptoms and lack of hemodynamic compromise, no intervention was required. The patient was counseled regarding the presence of this coronary venous anomaly and its potential clinical implications.

Particular attention was given to future procedures, including central venous catheter placement and cardiac device implantation, where altered venous anatomy may increase technical complexity and procedural risk. The incidental presence of PLSVC may further influence left-sided venous access routes.

The patient continues routine surveillance for his aortic disease, with no additional imaging required specifically for the venous anomaly.

## Discussion

The coronary venous system develops from the primitive subepicardial venous plexus, which progressively coalesces to form the major cardiac veins, including the great cardiac, middle cardiac, and left marginal veins [8-9]. These vessels ultimately drain into the coronary sinus, which is derived from the left horn of the sinus venosus and empties into the right atrium. The coronary sinus contains two valves: the Thebesian valve and the valve of Vieussens. The Thebesian valve, present in up to 86% of cases, is located at the junction of the coronary sinus and the right atrium and, when visualized, appears on computed tomography as a thin, hypodense linear structure [3].

Disruptions in this developmental process can result in a spectrum of anomalies, which have been broadly classified into four principal categories: (I) enlargement of the coronary sinus, (II) absence of the coronary sinus, (III) atresia of the right atrial coronary sinus ostium, and (IV) hypoplasia of the coronary sinus [10]. Persistent left superior vena cava (PLSVC) arises from failure of regression of the left anterior cardinal vein and frequently coexists with a dilated coronary sinus due to its drainage pattern [11]. While PLSVC is relatively common, associated abnormalities of coronary venous drainage are uncommon and reflect concurrent developmental variation in both systemic and cardiac venous structures [2].

Normal coronary sinus (CS) ostial dimensions have been described, with a median maximal diameter of 24 mm (IQR 19-30 mm) and a cross-sectional area of 87.9 mm² (IQR 56.5-127.1 mm²) reported by Alkhouli et al. However, unlike coronary artery disease, no standardized grading system exists for CS stenosis [12].

The hemodynamic effects of CS obstruction have been demonstrated experimentally. Elevated CS pressure can increase intramyocardial tissue pressure independently of coronary arterial pressure, particularly affecting subepicardial perfusion [13]. In this case, alternative drainage pathways, including direct drainage of the left marginal vein into the right atrium and a persistent left superior vena cava (PLSVC), likely acted as decompressive routes, preventing increased coronary venous pressure despite distal CS narrowing. This mechanism is supported by prior reports, where disruption of PLSVC flow resulted in myocardial congestion, while preservation of collateral pathways maintained hemodynamic stability [14-15].

Anomalous coronary venous drainage is typically asymptomatic and often identified incidentally, but it has important clinical implications due to its potential to complicate imaging interpretation and invasive procedures [5]. Altered venous pathways may increase technical difficulty during central venous access, cardiac device implantation, and cardiothoracic surgery [3-4]. Involvement of the coronary sinus may also have hemodynamic and electrophysiological consequences, including arrhythmias or conduction disturbances [16-17]. In more severe cases, such as coronary sinus ostial atresia, impaired venous drainage can lead to elevated coronary venous pressure, myocardial ischemia, or ventricular dysfunction [18]. Collateral pathways, thrombosis, and right-to-left shunting with risk of paradoxical embolization may also occur. Recognition of these anomalies is therefore essential to ensure accurate diagnosis and safe procedural planning [19].

In this study, atresia of the right atrial coronary sinus ostium in the setting of a persistent left superior vena cava (PLSVC) represents a rare deviation from normal venous anatomy, with only limited descriptions available [17]. Prior reports highlight the importance of detailed imaging evaluation, particularly with cardiac computed tomography, for accurate identification of such variants [2]. These anomalies are clinically relevant due to their hemodynamic and electrophysiological consequences, including arrhythmias and conduction disturbances. Impaired venous drainage may lead to elevated coronary venous pressure, myocardial ischemia, or ventricular dysfunction [16-17]. This finding underscores the need for systematic assessment of the coronary venous system, as recognition of rare drainage patterns is essential to avoid diagnostic pitfalls and to ensure safe and effective intervention and cardiac care management [3].

## Conclusions

Coronary sinus-related anomalous venous drainage is a rare entity with important implications for imaging interpretation, clinical correlation, and procedural planning. In this case, coronary sinus narrowing resulted in altered venous return with compensatory alternative drainage pathways, explaining the absence of symptoms despite significant anatomical variation. Recognition of these findings is essential to avoid misdiagnosis and inappropriate management. The presence of a persistent left superior vena cava represents an incidental and common variant and should be distinguished from the primary coronary sinus abnormality.
